# Associations of Candidate Gene Polymorphisms with Egg Production and Egg Quality Traits in Atak-S Laying Hens

**DOI:** 10.3390/ijms262412156

**Published:** 2025-12-18

**Authors:** Fatma Ilhan, Ali Aygun

**Affiliations:** Department of Animal Science, Faculty of Agriculture, Selcuk University, 42130 Konya, Turkey; aaygun@selcuk.edu.tr

**Keywords:** GH, IGF-1R, VIP12, egg quality, egg shape index

## Abstract

This study aimed to investigate the relationship between GH, GHR, IGF-1R, VIP, and NPY genes and egg quality traits in laying hens. Atak-S laying hens aged 54 weeks were monitored for 6 weeks. Egg production and egg weight were recorded daily, while egg quality traits and feed consumption were assessed weekly. Genotyping was performed using PCR-RFLP. The GH, GHR, IGF-1R, VIP, and NPY genes were cut with MspI, HindIII, HinfI, HinfI, and DraI, respectively. The AA genotype of the GH gene was associated with increased egg shape index, eggshell weight, and eggshell thickness (*p* < 0.05). In the IGF-1R region, significant associations were found with egg weight and egg shape index (*p* < 0.05). Additionally, the VIP12 TT genotype was linked to higher egg production (*p* < 0.05). These findings suggest that the GH gene may serve as a selection marker for shell-related traits, IGF-1R for egg weight and egg shape, and VIP for improving egg production. Overall, the results obtained in this study indicate that the genes studied have the potential to be candidate markers for improving egg performance and quality; however, their use in marker-assisted selection requires further studies in larger and more diverse populations.

## 1. Introduction

As the world’s population grows, the demand for protein increases day by day. Chicken meat and eggs, with their high protein and vitamin content, are important sources to meet this demand. With the technological developments in recent years, the poultry sector has taken on an important role in many countries. This increase has led to a rise in molecular studies in chickens. Identifying the relationship between molecular markers and economically important yield traits, and selecting for these markers, can significantly enhance the efficiency of animal production while reducing the time required for genetic improvement. By using molecular markers as indicators of desirable traits, breeders can make more precise and faster selection decisions, leading to improved productivity, higher-quality products, and better resource management in animal breeding programs.

Many imported commercial breeds of broilers and laying hens are used in Turkey. Although the use of these breeds contributes to the country’s poultry industry, it has also led to the occurrence of various diseases (C.R.D., Marek’s disease, laryngotracheitis, and Gumboro) and created dependence on imported breeding material [1]. It has been observed that Atak-S laying hens have a stronger immune system compared to other commercial breeds. The ATAK-S genotype is a laying genotype developed in Turkey. This genotype is used in both cage systems [2,3] and free-range systems [4,5]. However, its productivity is lower than that of imported lines, and therefore, genetic improvement of this breed is a priority for the sustainability of the national poultry industry.

Molecular markers play a crucial role in enhancing animal productivity traits through marker-assisted selection. Identifying associations between molecular markers and traits such as egg production and quality can significantly improve breeding efficiency and accelerate genetic progress. Several candidate genes, including GH, GHR, IGF-1, IGF-1R, VIP, and NPY, have been reported to influence growth, reproduction, and egg production in poultry [6,7,8]. In laying hens, these genes—particularly growth hormone (GH), insulin-like growth factor (IGF)-I, gonadotropin-releasing hormone (GnRH), prolactin (PRL), vasoactive intestinal polypeptide (VIP), and neuropeptide Y (NPY) are key regulators of egg production and quality [9]. Commonly analyzed using PCR-RFLP methods, these genes have been linked to important economic traits in poultry through marker-assisted selection studies [10].

GH, its receptor (GHR), and IGF-related genes play central roles in growth, metabolism, and reproduction. GH, produced by the anterior pituitary gland, influences growth, egg production, and immunity, while GHR, located on the Z chromosome, mediates GH activity [7]. The IGF-1 gene, situated on chromosome 1, regulates growth and ovarian follicle development, and its receptor, IGF-1R, contributes to muscle development and metabolic control. The VIP gene (chromosome 3) affects prolactin secretion and reproductive behavior, with certain polymorphisms associated with egg number and shell quality [11].

Likewise, the NPY gene (chromosome 2) influences feed intake, reproduction, and energy metabolism, potentially affecting sexual maturity and laying performance [12]. Collectively, these genes represent valuable molecular markers for improving productivity and reproductive traits in laying hens [13].

While the IGF-1R gene has been studied for its relationship with growth and carcass traits, there are insufficient studies on its association with egg production and quality. Similarly, the roles of all the genes examined in this study in growth, reproduction, and endocrine regulation have been established. However, information on their involvement in egg production and quality is limited. These genes were selected because their biological importance has been highlighted in previous studies, they have been proposed as candidate genes for marker-assisted selection, and they are believed to be associated with egg production and quality. Therefore, this study aimed to determine the allelic and genotypic distributions of the GH, GHR, IGF-1R, VIP, and NPY genes in Atak-s chickens for polymorphisms in these genes and to evaluate the relationship of these genotypes with egg performance and quality.

## 2. Results

### 2.1. PCR-RFLP Analysis

**Figure 1:**
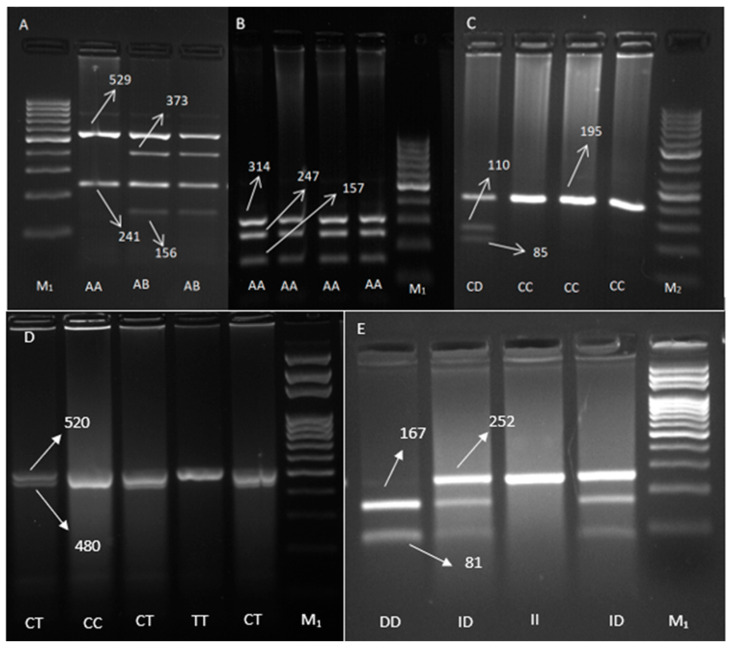
PCR-RFLP pattern of for GH gene with Msp1 enzyme (**A**), GHR gene with HindIII enzyme (**B**), IGF-1R gene with Hinf1 enzyme (**C**), VIP12 gene with Hinf1enzyme (**D**), NPY gene with DraI enzyme (**E**). Lane M1: 100 bp DNA ladder marker for (**A**,**B**,**D**,**E**) figure. Lane M2: 50 bp DNA ladder Marker for (**C**) figure.

The enzymes used to cut the studied gene regions, the sequences recognized by these enzymes, and the genotypes obtained after cutting are listed in Table 1. The gel images obtained after enzyme cutting of the gene regions used in the study are shown in Figure 1. A 770 bp fragment of the first intron of the GH gene was amplified by PCR, and two genotypes (AA and AB) were obtained after cutting with the MspI enzyme. The BB genotype was not observed in the population used in the study. The 718 bp exon 2 region of the GHR gene was amplified and cut with the HindIII enzyme; however, the AA genotype was observed in all birds. The 195 bp sequences of the 1st (first) intron of the IGF-1R gene were amplified and cut with the Hinf1 enzyme, and two genotypes (CC and CD) were determined. The 520 bp portion of the second intron region of the VIP12 gene was amplified and cut with the Hinf1 enzyme, yielding three genotypes (TT, CT, and CC). Finally, the 252 bp portion of the NPY gene at the transcription start site was cut with the DraI enzyme and three genotypes (II, ID, and DD).

The allele and genotype frequencies are listed in Table 2. According to the results of the chi-square analysis, it was found that the studied population was not in equilibrium (*p* < 0.05) for gene regions other than VIP12. The heterozygosity in all studied gene regions was higher than expected.

### 2.2. Descriptive Statistics and Correlation Analysis of Performance and Egg Quality Traits

Descriptive statistics and correlation analyses were conducted to evaluate the performance and egg quality characteristics of Atak-S laying hens. Mean values, variability indicators, and relationships among major traits were examined to assess overall production uniformity and inter-trait associations. The results are presented in Table 3.

The average egg weight was 61.78 ± 3.53 g, with a minimum of 52.06 g and a maximum of 69.75 g. Egg mass averaged 56.24 ± 5.86 g/hen per day, while egg production was 91.39 ± 8.45%. Feed intake ranged from 104.93 to 159.9 g/hen per day, with a mean of 133.31 ± 12.02 g, and the feed conversion ratio averaged 2.11 ± 0.26 g feed/g egg.

Regarding egg quality traits, the mean eggshell breaking strength was 3.02 ± 0.006 kg, eggshell weight 5.19 ± 0.52 g, and eggshell thickness 0.41 ± 0.04 mm. Internal quality parameters showed a mean Haugh Unit of 87.48 ± 5.46, an albumen index of 6.99 ± 1.49%, a yolk index of 41.55 ± 1.98%, and an egg shape index of 73.84 ± 2.72%. Coefficients of variation (CV) for all traits were within acceptable limits, indicating consistent performance and uniformity among the measured parameters.

The correlations among performance traits are presented in Table 4. Egg mass was positively and significantly correlated with egg weight (r = 0.484, *p* < 0.001) and egg production (r = 0.849, *p* < 0.001). Feed intake also showed a moderate positive correlation with egg weight (r = 0.341, *p* = 0.003) and egg mass (r = 0.350, *p* = 0.003). In contrast, feed conversion ratio exhibited strong negative correlations with egg mass (r = −0.798, *p* < 0.001) and egg production (r = −0.772, *p* < 0.001), indicating that hens with higher productivity had better feed efficiency. These results demonstrate that increased egg production and mass are associated with improved feed utilization efficiency.

Significant positive correlations were observed among several egg quality traits (Table 5). Eggshell breaking strength showed a strong positive correlation with eggshell weight (r = 0.511, *p* < 0.001) and thickness (r = 0.492, *p* < 0.001). A very high correlation was found between albumen index and Haugh unit (r = 0.843, *p* < 0.001). In contrast, other correlations among yolk and albumen indices were generally weak or non-significant. These findings indicate that eggshell characteristics are closely related to each other, while internal egg quality traits, such as albumen height and Haugh unit, are strongly interdependent.

### 2.3. Association of Genotypes with Egg Quality Traits

The results of the analysis conducted to determine the relationship between the performance and egg quality traits across all gene regions included in the study are shown in Table 6 and Table 7. Since two genotypes were obtained by enzyme editing of GH and IGF-1R genes, a t-test was performed. Additionally, as three genotypes were obtained by editing the VIP12 and NPY genes, an ANOVA test was conducted to assess the relationships. It was found that the quality traits of the GH genotypes were associated with the eggshell breaking strength, egg shape index (*p* < 0.05), eggshell weight, and eggshell thickness (*p* < 0.05). Eggs from hens with the AA genotype exhibited higher values for these traits, while no statistically significant difference was observed between genotypes for other traits.

The relationship between the genotypes obtained by cutting the IGF-1R gene region with the Hinf1 enzyme and egg quality traits was assessed. This gene region showed a significant relationship between IGF-1R genotypes and egg weight as well as egg shape index (*p* < 0.05), but it was not associated with other traits. Birds with the CC genotype produced heavier eggs, while the eggs from birds with the CD genotype had a higher egg shape index. Additionally, a correlation was found between the genotypes obtained by cutting the VIP12 gene with the Hinf1 enzyme and egg yield (*p* < 0.05). The percentage of egg yield was higher in hens with the TT genotype compared to other genotypes.

There was no statistically significant correlation between the genotypes obtained by DraI enzyme cut of the NPY gene and hen performance and egg quality.

## 3. Discussion

Egg production is an important trait in laying hens, controlled by numerous genes and significantly influenced by the environment. Due to its sensitivity to environmental factors, increasing egg production is difficult using conventional breeding methods. Therefore, identifying the complex genetic structure underlying egg production is crucial for increasing egg yield and improving breeding programs [14].

Therefore, identifying genes associated with egg production is a crucial step in the efficient breeding of laying hens. In this context, this study aimed to determine the relationship between egg production, egg quality traits in the domestic breed Atak-s laying hens, and the GH, GHR, IGF, VIP12, and NPY genes. PCR-RFLP analysis identified two genotypes for the GH and IGF-1R genes and three genotypes for the VIP12 and NPY genes.

The results obtained for egg production performance are generally consistent with the literature. The average egg weight in this study was 61.78 g, which is similar to the egg weights of 60–62 g reported by Alig et al. [15] and Darmawan et al. [16]. Average feed consumption (133.31 g/hen/day) and feed conversion ratio (2.11 g feed/g egg) are also comparable to the values reported by Alfonso-Carrillo et al. [17].

When egg quality parameters were examined, shell fracture resistance (3.12 kg) and shell thickness (0.41 mm) were found to be within optimum limits, indicating balanced calcium metabolism [18]. The shape index and Haugh unit were calculated as 74.84 and 87.48, respectively, which are consistent with the values reported by Saleh et al. [19] and Esenbuga and Ekinci [20].

Pearson correlation analysis revealed a positive correlation between egg weight and egg mass, due to the direct dependence of egg mass on egg weight. The positive correlation between egg production rate and egg mass indicates that increased production also increases daily egg mass. Weak to moderate positive correlations were found between feed intake and both egg production and egg mass, demonstrating that nutrient intake supports production performance. Conversely, negative correlations were found between feed conversion ratio and both egg production and egg mass, indicating that increased yields improve feed utilization efficiency.

Positive and significant correlations were found between eggshell breaking strength, shell weight, and shell thickness (*p* < 0.001). This effect can be explained by the increase in eggshell breaking strength as mineral accumulation and structural integrity improve. Similarly, the relationship between shell thickness and shell weight was also positive, confirming that these traits are important determinants of shell quality. The egg shape index showed weak positive correlations with the albumen and yolk indices. The positive and significant correlation between the Haugh unit and yolk index suggests that internal egg quality is related to freshness and albumen content.

In order to evaluate the relationship between phenotypic data and genes, the GH gene region was first evaluated. Two genotypes were obtained for the GH gene through MspI enzyme cut. The eggshell breaking strength, egg shape index, eggshell weight, and eggshell thickness were higher in hens with the AA genotype in the GH gene region. In studies conducted to uncover the association between the GH gene region and egg production, GH genotypes were found to be associated with traits such as first egg weight, egg production, and age at first laying [6]. Eggshell quality is largely influenced by environmental factors such as nutrition, age, and ambient temperature; however, previous research has demonstrated that genetic factors also play a significant role. In a genome-wide association study (GWAS) conducted by Sun et al. [21], numerous single-nucleotide polymorphisms (SNPs) located in three different genes were identified to be associated with eggshell quality. Notably, one of these SNPs was found to be related to eggshell calcification and, consequently, may influence eggshell breaking strength. Similarly, studies conducted by Dunn et al. [22] have shown that SNPs located in various genes may be associated with eggshell breaking strength. Similarly, in our study, a significant association was observed between GH genotypes and eggshell breaking strength. Eggs from hens carrying the AA genotype exhibited higher breaking strength compared to those from hens with the AB genotype, indicating that GH genotypes have a measurable effect on eggshell breaking strength. Studies in various animal species indicate that GH can increase intestinal calcium absorption, either directly or via IGF-1 [23]. The discovery of a similar mechanism in poultry suggests that GH may be related to eggshell formation and, consequently, shell quality.

The egg shape index is one of the values used to determine egg quality. The egg shape index, defined as the ratio of egg width to egg length, is an important criterion for assessing egg quality [24]. Round eggs have a weaker appearance and are less suitable for egg trays. During transportation, these eggs are more prone to breakage and cracking compared to normally shaped eggs [25]. A study conducted by Sekeroglu et al. [26] found a correlation between the egg shape index and various quality traits, including eggshell thickness, albumen length, yolk width, yolk height, and yolk color. Studies have shown that the egg shape index is related to hatching traits such as chick survival rate [10], hatching rate from fertilized eggs, and early embryonic mortality rate [27]. Alasahan and Copur [27] found that chickens hatched from eggs with a small shape index had a higher carcass weight. The quality characteristics of the eggshell are among the most important criteria in egg production. Poor shell quality leads to economic losses during transportation and storage of the eggs. The correlation of GH genotypes with eggshell weight and eggshell thickness suggests that the GH gene region can be used as a selection marker in relation to egg quality criteria.

Two genotypes were determined by cutting the IGF-1R gene region using the Hinf1 enzyme. Although the relationship between genotypes in this gene region and meat yield in chickens has been discussed in some studies [28], the relationship with egg production has not been thoroughly investigated. In this regard, this study is pioneering. The IGF-1R gene region was found to be associated with egg weight and egg shape index. A high egg weight is preferred in egg production, as heavier eggs can be sold at higher prices, leading to more profitable production. The IGF-1R gene has been reported to be associated with ovarian follicle development and reproductive endocrinology. This association suggests that IGF-1 receptors are distributed throughout theca and granulosa cells, stimulating hormone secretion and follicle development [29,30]. This mechanism may influence egg weight.

Three genotypes were obtained by cutting the Hinf1 enzyme in the VIP12 gene: TT, CT, and CC, similar to previous studies [31,32,33]. A significant correlation was found between the genotypes obtained and the performance traits, only for egg production (*p* < 0.05). Table 4 shows that, on average, the percentage egg yield is higher in birds with the TT genotype. Similarly to this study, in another PCR-RFLP study conducted on the second intron of the VIP gene, egg productivity of birds with the TT genotype was found to be high [32]. Zhou et al. [31] found in their study on chickens that there is a relationship between VIP genotypes and the number of eggs. The VIP gene activates signalling pathways that affect gonadotropin synthesis and ovarian follicle development, which in turn influence ovulation and its frequency [9]. Given this mechanism, it is plausible that VIP genotypes may affect egg production. However, several other studies have found that VIP genotypes are not associated with egg production and quality [11,34], suggesting that the effect of VIP may vary depending on population structure, management conditions, or genetic background.

For the NPY gene, the other gene included in the study, three genotypes (II, ID, DD) were identified, similar to previous studies [7,35,36]. Statistical analysis revealed that there was no statistically significant association between NPY genotypes and laying hen performance and egg quality. A study on the NPY gene in laying hens found that chickens with the ID genotype exhibited higher daily weight gain and greater body weight [37]. Similarly, a study on local Iraqi chicken breeds reported that the 100-day egg production was higher in chickens with the NPY/II genotype [36]. In another study, Promket et al. [12] determined that hens with the NPY/DD genotype had lower egg production and egg mass on day 270. Several other studies conducted in this gene region concluded that egg production and NPY genotypes are related [12,37,38]. The absence of a relationship between the NPY gene and egg production and quality in this study may be due to the evaluation of egg production criteria at different times during the laying period. Additionally, differences in statistical methods and environmental factors may also have contributed.

Another important finding regarding the evaluation of genotype distributions is the Hardy–Weinberg equilibrium analysis. These traits deviated from Hardy–Weinberg equilibrium at all locations except VIP 12. This result is expected because Atak-S chickens were produced by crossbreeding commercial breeds, so they were not formed through natural selection by random mating. It is possible that the resulting groups, when mated with different groups, deviated from equilibrium. Similarly, the GHR locus was found to be monomorphic in this population. As previously mentioned, the findings may be due to the limited genetic diversity resulting from the Atak-S chicken background; intense selection and the use of narrow parental lines during this period may have led to the disappearance of certain alleles while others persisted. Consequently, a single allele may have remained, causing the GHR gene to appear monomorphic.

When the findings are evaluated in general, it is thought that these gene regions may have an effect on egg production and quality traits. This study provides valuable insights into the relationships among egg production, egg quality traits, and specific gene regions. In particular, the GH, IGF-1R, and VIP12 gene regions were identified as significant factors influencing egg production and egg quality. The incorporation of these gene regions into selection programs could serve as an effective strategy to enhance production efficiency. It is anticipated that this study will contribute to future genetic selection and breeding programs.

## 4. Materials and Methods

### 4.1. Experimental Population and Phenotypic Measurements

The Atak-s laying hens are a high-performance hybrid derived from the crossbreeding of Rhode Island Red and Barred Plymouth Rock, developed at the Poultry Research Institute in Turkey [39]. This chicken line has been maintained and improved for many years through systematic selection programs aimed at enhancing egg yield, persistency, and overall adaptation to local production conditions. As a national hybrid developed in Turkey, Atak-S plays a significant role in the country’s egg production sector by reducing dependency on imported commercial breeds and by demonstrating strong adaptability to organic and free-range farming systems. Therefore, the continued genetic and phenotypic improvement of the Atak-S laying hens remains essential. Molecular studies on this hybrid will further support its productivity and contribute to more economically efficient outcomes.

A total of 72 Atak-S laying hens were randomly selected from the flock maintained at Selçuk University, Faculty of Agriculture, Department of Animal Sciences. Although detailed pedigree information for the Atak-S hybrid is proprietary and not publicly accessible, the birds housed at Selçuk University originate from the official multi-line Atak-S breeding program conducted by the Poultry Research Institute. Therefore, the sampled hens represent the current genetic background of the Atak-S production population.

The hens were randomly placed into individual cages at 16 weeks of age to allow sufficient time for cage adaptation before data collection. The hens remained housed in these cages until the start of the experiment. All egg production and egg quality measurements were initiated when the hens reached 54 weeks of age. Each cage was 30 × 50 cm and was arranged in a three-tier system, equipped with one nipple drinker per cage. The lighting schedule was automatically set to provide 16 h of light and 8 h of darkness. A constant temperature of 20 °C and a relative humidity of 50% were maintained in the poultry house to ensure optimal environmental conditions. The hens were fed a commercial laying hen diet sourced from a reputable feed manufacturer. The feed was finely ground and offered ad libitum. It contained 17% crude protein, 2700 kcal/kg metabolizable energy (ME), 4% calcium (Ca), and 0.45% available phosphorus (P), ensuring a balanced nutrient intake for optimal egg production.

Egg production and egg weight were determined daily, while egg quality traits and feed consumption were assessed weekly. In total, an average of 40 eggs from each chicken were weighed and 6 eggs from each chicken were used for egg quality analysis. Egg quality traits were measured on all eggs collected in the last two days of each week, and eggshell breaking strength, eggshell thickness, eggshell weight, and internal egg quality traits were calculated. The egg shape index (%) = [egg width (mm)/egg length (mm)] × 100 was calculated using the formula. The height of the yolk and egg yolk was determined using a digital caliper (Mitutoyo Inc., Kawasaki, Japan) and the diameter of the yolk and egg yolk was measured using a digital caliper (Mitutoyo Inc., Kawasaki, Japan). Yolk index (%) = (yolk height/yolk diameter) × 100, Albumen index (%) = (albumen height/((albumen length + albumen width)/2)) × 100. Haugh unit = 100 × log (albumen height + 7.57 − 1.7 × egg weight ^0.37^) [40]. The eggshell breaking strength was measured using an Egg Force Reader device (Orka Food Tech., Hong Kong, China). The eggs were then broken, and the shell, albumen, and yolk were separated and weighed individually. The eggshell was carefully washed and air-dried to remove the shell membrane and then weighed [41]. The eggshells were weighed using a precision balance with an accuracy of 0.001 g. The thickness of the eggshell, including the membrane, was measured at three points on each egg (blunt end, equatorial region, and sharp end) using a micrometer (Mitutoyo Inc., Kawasaki, Japan) [42].

### 4.2. DNA Isolation and PCR-RFLP Analysis

At the end of the experiment, blood was collected from the brachial vein of all chickens included in the study for DNA isolation. At least 1 mL of blood was obtained from each hen in a single sampling. The blood samples collected from all 72 hens were preserved under cold chain conditions and stored in the laboratory until DNA isolation. Total DNA was extracted from the blood samples using the salting-out method [43]. DNA samples were analyzed on a 1% agarose gel to determine DNA concentration and purity. DNA isolation was repeated for samples that did not exhibit visible bands on the gel. Following DNA isolation, the target gene regions of the samples were amplified by PCR. The primers used for amplifying the target gene regions are listed in Table 5.

For 10 µL PCR solution, 2 µL DNA (50–100 ng/µL), 0.25 µL (10 pmol/µL) of each primer, 5 µL Taq green 2 × PCR Master Mix (50 mM Tris-HCl (pH 9.0 at 25 °C), 50 mM NaCl, 5 mM MgCl_2_, 200 μM each of dATP, dCTP, dGTP, dTTP) and 2.5 µL ddH_2_O (Sterile Double Distilled Water) were used. The PCR protocol consisted of an initial denaturation step at 94 °C for 5 min, followed by 35 cycles of denaturation at 94 °C for 30 s, annealing at the temperature indicated in the table for 45 s, and an extension step at 72 °C for 1 min. The PCR process was completed with a final hold at 72 °C for 10 min. After PCR, samples were analyzed on a 1% agarose gel containing ethidium bromide. Restriction fragment length polymorphism (RFLP) analysis was performed to identify polymorphism at the locus of interest. Restriction enzymes (Thermo Scientific Inc., Waltham, MA, USA) were chosen according to the studies listed in Table 8 and subsequently used for genotyping the target gene regions. Approximately 10 µL of the PCR product was mixed with 2 µL of buffer, 0.5 µL of restriction enzyme, and 7.5 µL ddH_2_O, and incubated overnight at 37 °C. The RFLP products were separated by agarose gel electrophoresis (2%) in 1 × TE buffer containing ethidium bromide. Ethidium bromide is a fluorescent dye that binds to DNA and is used to visualize DNA bands under UV light.

The numbers of eggs used for egg weight and egg quality analyses for each genotype are presented in Table 9.

### 4.3. Statistical Analysis

Descriptive statistics and correlation analyses were performed to summarize and evaluate the relationships between egg production and quality traits of Atak-S laying hens. POPGEN 1.32 software was utilized to calculate allele and genotype frequencies, chi-square values, and heterozygosity metrics. Since there were two genotypes for GH and Igf-1R, an independent samples t-test was conducted using SPSS v.25.0. For VIP and NPY, which involved three genotypes, a one-way ANOVA test was performed to assess statistical differences among the groups. The statistical model used for the ANOVA is presented below.

Yij = µ + ai+ eij

Yij: Trait value,

μ: Population mean,

ai: The effect of genotype,

eij: Random error

For egg quality traits (e.g., egg weight, shell thickness, shell strength), all measurements collected from each hen during the six-week experimental period were averaged to obtain a single representative value per hen. These per-hen mean values were subsequently used for genotype comparisons (e.g., AA vs. AB) through one-way ANOVA. This approach focuses on testing the primary hypothesis concerning the overall effect of genotype, while reducing the dependency issue that arises from repeated measurements within the same hen.

The phenotypic results obtained were grouped by genotype, and the mean ± standard error mean was calculated. The association between each trait and genotype was determined using appropriate statistical analysis. Each trait was analyzed independently using phenotypic measurements.

## 5. Conclusions

This study aimed to determine the relationship between GH, GHR, IGF-1R, VIP12, and NPY genes and egg performance and egg quality traits. The analysis revealed that the genotypes of GH, IGF-1R, and VIP12 are associated with egg production and egg quality traits. It is hypothesized that the GH gene region can be used as a selection criterion in breeding programs concerning egg shape index, eggshell weight, and eggshell thickness. Similarly, the IGF-1R gene region may serve as a selection criterion for egg weight and egg shape index. This study contributes to the limited existing evidence regarding the relationship between the IGF-1R gene region and egg quality traits in laying hens. The VIP gene can be considered in studies aimed at increasing egg productivity. These results provide preliminary evidence that these genes may influence egg-related traits, but their value as selection markers remains to be confirmed through larger-scale and multi-generational studies.

## Figures and Tables

**Table 1:** Some descriptive information of studied candidate genes.

Locus	Region	Length	Restriction Enzyme	Restriction Site	Genotypes	bp
GH	Intron 1	770	MspI	5′-C/CGG-3′	AA	529/241
					AB	529/373/241/156
GHR	Exon 2	718	HindIII	5′-A/AGCTT-3′	AA	314/247/157
IGF-1R	Intron 1	195	Hinf1	5′-G/ANTC-3′	CC	195
					CD	195/110/85
VIP12	Intron 2	520	Hinf1	5′-G/ANTC-3′	TT	520
					CT	520/480/40
					CC	480/40
NPY	Transcription Start Site	252	DraI	5′-TTT/AAA-3′	II	252
					ID	252/167/81
					DD	167/81

**Table 2:** Allele and genotype frequencies of candidate genes.

Gene	Allele Frequencies			Genotypes Frequencies (n)	Ho	He	χ^2^
GH	A	0.59	AA (13)	0.18	0.82	0.49	34.03 *
	B	0.41	AB (59)	0.82			
			BB (0)	0			
IGF-1R	C	0.76	CC (40)	0.55	0.48	0.37	5.06 *
	D	0.24	CD (32)	0.45			
			DD (0)	0			
VIP12	T	0.75	TT (40)	0.55	0.42	0.37	1.08
	C	0.25	CT (30)	0.41			
			CC (2)	0.04			
NPY	I	0.49	II (10)	0.14	0.69	0.50	7.00 *
	D	0.51	ID (50)	0.69			
			DD (12)	0.17			

$\chi_{0.05;1}^{2}$: 3.84 * Deviation from HWE is significant. Ho; observed heterozygosity, He; expected heterozygosity.

**Table 3:** Descriptive statistics of egg quality and performance traits in Atak-S hens.

	Mean	Std Dev	Min	Max	CV
Egg weight (g)	61.78	3.53	52.06	69.75	5.71
Egg mass (g/hen per day)	56.24	5.86	32.10	69.29	10.61
Egg Production (%)	91.39	8.45	60	100	9.34
Feed Intake (g/hen per day)	133.31	12.02	104.93	159.9	9.09
Feed conversion ratio (g feed/g egg)	2.11	0.26	1.61	3.07	12.2
Eggshell breaking strength (kg)	3.121	0.006	1.601	4.610	19.03
Egg Shape index (%)	74.84	2.72	67.22	84.18	3.68
Albumen index (%)	6.99	1.49	4.32	11.14	21.32
Yolk index (%)	41.55	1.98	37.11	46.57	4.77
Haugh Unit	87.48	5.46	69.65	98.9	6.24
Eggshell weight (g)	5.19	0.52	3.7	6.6	10.04
Eggshell thickness (mm)	0.41	0.04	0.3	0.51	9.88

**Table 4:** Correlation coefficients among performance traits in Atak-S hens.

	EW	EM	EP	FI
EM	0.484 ** (0.000)			
EP	−0.038 (0.749)	0.849 ** (0.000)		
FI	0.341 ** (0.003)	0.350 ** (0.003)	0.212 (0.074)	
FCR	−0.224 (0.059)	−0.798 ** (0.000)	−0.772 ** (0.000)	0.238 * (0.044)

The Pearson correlation coefficients and *p* values (in parentheses) are shown within the cells (** *p* < 0.01; * *p* < 0.05); EW: Egg weight (g), EM: Egg mass (g/hen per day), EP: Egg production (%), FI: Feed Intake, FCR: Feed conversion ratio (g feed/g egg).

**Table 5:** Correlation coefficients among egg quality traits in Atak-S hens.

	EBS	ESI	AI	YI	HU	ESW
ESI	0.236 * (0.046)					
AI	0.172 (0.167)	0.046 (0.713)				
YI	0.138 (0.250)	0.304 * (0.010)	0.515 ** (0.000)			
HU	0.189 (0.115)	0.118 (0.328)	0.843 ** (0.000)	0.430 ** (0.000)		
ESW	0.511 ** (0.000)	0.222 (0.103)	−0.095 (0.510)	−0.009 (0.951)	−0.009 (0.947)	
EST	0.492 ** (0.000)	0.235 (0.055)	0.089 (0.494)	0.098 (0.432)	0.099 (0.431)	0.370 ** (0.007)

The Pearson correlation coefficients and *p* values (in parentheses) are shown within the cells (** *p* < 0.01; * *p* < 0.05); EBS: Eggshell breaking strength (kg) ESI: Egg shape index (%), AI: Albumen index (%), YI: Yolk index (%), HU: Haugh Unit, ESW: Eggshell weight (g), EST: Eggshell thickness (mm).

**Table 6:** The results of the performance traits determined according to genotyping.

	Genotypes	Egg Weight (g)	Egg Mass (g/Hen per Day)	Egg Production (%)	Feed Intake (g/Hen per Day)	Feed Conversion Ratio (g Feed/g Egg)
GH	AA	61.67 ± 1.10	57.53 ± 1.90	93.15 ± 1.72	133.7 ± 2.42	2.04 ± 0.065
	AB	61.04 ± 0.65	55.02 ± 0.94	90.20 ± 1.45	132.5 ± 1.20	2.13 ± 0.042
IGF-1R	CC	62.58 ± 0.60 ^a^	56.64 ± 0.84	91.51 ± 1.33	132.9 ± 1.87	2.06 ± 0.035
	CD	60.75 ± 0.65 ^b^	54.09 ± 1.40	89.80 ± 1.90	132.3 ± 1.62	2.18 ± 0.061
VIP12	TT	61.69 ± 0.78	56.25 ± 0.91	92.50 ± 1.52 ^a^	133.6 ± 1.94	2.09 ± 0.041
	CT	61.61 ± 0.65	54.46 ± 1.34	89.06 ± 1.78 ^b^	132.2 ± 1.81	2.16 ± 0.049
	CC	62.96 ± 0.52	55.30 ± 1.13	88.33 ± 1.26 ^b^	132.8 ± 1.24	2.15 ± 0.044
NPY	II	60.82 ± 0.83	56.57 ± 1.13	94.05 ± 2.13	128.1 ± 1.76	1.97 ± 0.029
	ID	61.59 ± 0.50	55.29 ± 1.03	93.78 ± 1.45	133.0 ± 1.49	2.13 ± 0.045
	DD	60.36 ± 1.41	57.12 ± 1.89	95.48 ± 1.72	136.1 ± 1.95	2.09 ± 0.067

^a,b^: Means within a column with different superscripts differ significantly (*p* < 0.05).

**Table 7:** The results of the egg quality traits determined according to genotyping.

Gene	Genotypes	Eggshell Breaking Strength (kg)	Egg Shape Index (%)	Albumen Index (%)	Yolk Index (%)	Haugh Unit	Eggshell Weight (g)	Eggshell Thickness (mm)
GH	AA	3.36 ± 0.162 ^a^	77.7 ± 1.31 ^a^	7.20 ± 0.612	41.71 ± 0.70	89.1 ± 1.90	5.47 ± 0.140 ^a^	0.352 ± 0.021 ^a^
	AB	2.99 ± 0.086 ^b^	73.6 ± 0.33 ^b^	7.06 ± 0.231	41.67 ± 0.28	87.8 ± 0.81	5.11 ± 0.088 ^b^	0.323 ± 0.008 ^b^
IGF-1R	CC	2.96 ± 0.096	73.6 ± 0.47 ^b^	7.01 ± 0.294	41.47 ± 0.32	87.4 ± 1.02	5.26 ± 0.091	0.315 ± 0.022
	CD	3.08 ± 0.116	76.8 ± 0.46 ^a^	7.11 ± 0.302	41.64 ± 0.38	88.3 ± 1.05	5.18 ± 0.903	0.318 ± 0.010
VIP12	TT	3.20 ± 0.124	73.6 ± 0.46	6.97 ± 0.330	41.25 ± 0.60	86.9 ± 1.31	5.02 ± 0.121	0.324 ± 0.012
	CT	3.15 ± 0.136	73.7 ± 0.41	7.19 ± 0.301	41.90 ± 0.31	88.3 ± 0.92	5.32 ± 0.113	0.334 ± 0.009
	CC	3.80 ± 0.120	76.9 ± 0.50	5.94 ± 0.326	40.31 ± 0.42	85.6 ± 0.95	5.16 ± 0.092	0.342 ± 0.002
NPY	II	3.20 ± 0.283	73.1 ± 0.82	7.03 ± 0.682	40.88 ± 0.724	88.2 ± 2.24	5.02 ± 0.320	0.333 ± 0.028
	ID	3.02 ± 0.104	74.4 ± 0.51	6.92 ± 0.244	42.10 ± 0.310	87.6 ± 0.94	5.25 ± 0.092	0.334 ± 0.010
	DD	2.91 ± 0.233	73.2 ± 0.93	8.11 ± 0.982	41.14 ± 0.753	88.5 ± 2.76	4.98 ± 0.212	0.309 ± 0.023

^a,b^: Means within a column with different superscripts differ significantly (*p* < 0.05).

**Table 8:** Lengths, primer sequences and annealing temperatures of the studied gene regions.

Locus	Primer Sequence	Length	Annealing Temp (°C)	References
GH	F5′-ATCCCCAGGCAAACATCCTC-3′ R5′-CCTCGACATCCAGCTCACAT-3′	770	62	[44]
GHR	F5′-GGCTCTCCATGGGTATTAGGA-3′ R5′-GCTGGTGAACCAATCTCGGTT-3′	718	59	[45]
IGF-1R	F5′-GAGCCTGCACAGACCAGAAT-3′ R5′-CAGGGACTTTGGAGCAGAAC-3′	195	58	[46]
VIP12	F5′-GCTTGGACTGATGCGTACTT-3′ R5′-GTATCACTGCAAATGCTCTG-3′	520	58	[31]
NPY	F5′-TCTCAGAGCTCCAACGTATGA-3′ R5′-ATATTTCTGTGCCTGAACAACA-3′	252	57	[7]

**Table 9:** Number of Eggs Used for Egg Weight and Egg Quality Analyses.

Gene	Genotype	Eggs Used for Egg Weight	Eggs Used for Egg Quality Analysis
GH	AA	519	78
	AB	2328	354
IGF	CC	1527	240
	CD	1280	192
VIP	TT	1512	240
	CT	1342	180
	CC	87	12
NPY	II	432	60
	ID	2124	300
	DD	483	72

## Data Availability

The datasets are available from the corresponding author upon reasonable request.

## Abbreviations

The following abbreviations are used in this manuscript:

GH	Growth Hormone
GHR	Growth Hormone Receptor
IGF-1R	Insulin-like Growth Factor 1 Receptor
VIP	Vasoactive Intestinal Polypeptide
NPY	Neuropeptide Y
PCR	Polymerase Chain Reaction
RFLP	Restriction Fragment Length Polymorphism
kb	Kilobase
bp	Base Pair
ANOVA	Analysis of Variance
MAS	Marker-Assisted Selection
ddH_2_O	Double Distilled Water
TE buffer	Tris-EDTA buffer used for preserving DNA
SPSS	Statistical Package for the Social Sciences
Ho	Observed Heterozygosity
He	Expected Heterozygosity
ME	Metabolizable Energy
Ca	Calcium
P	Phosphorus
HWE	Hardy–Weinberg Equilibrium

## References

[1] Özdemir S., Arslan H., Özentürk U., Yıldırım F., Yıldız A. (2018). Estimated genetic diversity between Atak-S and Isa Brown chickens with SSR markers. Kocatepe Vet. J..

[2] Gök İ., Kurşun K. (2025). Comparative Estimation Models of Egg Albumen Index in Atak-S Hens with Ridge and Principal Component Regression Methods. Turk. J. Agric. Food Sci. Technol..

[3] Yılmaz Dikmen B., Gündüz M., Kaşif A., Sevinç B.F. (2025). Determination of Genotype, Housing System and Age Effect on Egg Production and Quality Traits of Layers. J. Poult. Res..

[4] Sözcü A., İpek A., Oguz Z., Gunnarsson S., Riber A.B. (2021). Comparison of performance, egg quality, and yolk fatty acid profile in two Turkish genotypes (Atak-S and Atabey) in a free-range system. Animals.

[5] Aygun A., Narinç D., Arısoy H. (2025). Comparison of Performance, Egg Quality, and Egg Cost of Different Laying Genotypes in Free-Range System from 21 to 44 Weeks of Age. Animals.

[6] Kazemi H., Rezaei M., Hafezian H., Mianji G., Najafi M. (2018). Genetic analysis of SNPs in GH, GHR, IGF-I and IGFBPII genes and their association with some productive and reproductive traits in native breeder hens. Gene Technol..

[7] Wu X., LI H.F., Yan M.J., Tang Q.P., Chen K.W., Wang J.Y., Gao Y.S., Tu Y.J., Yu Y.B., Zhu W.Q. (2007). Associations of gonadotropin-releasing hormone receptor (GnRHR) and neuropeptide Y (NPY) genes’ polymorphisms with egg-laying traits in Wenchang chicken. Agric. Sci. China.

[8] Ruangwittayanusorn K., Promket D., Pimrueng K., Kammongkun J. (2022). The association of dopamine receptor D2 (DRD2) and vasoactive intestinal peptide (VIP) polymorphisms on egg production in high egg strain of pradu hangdum chiangmai chickens. Adv. Anim. Vet. Sci..

[9] Wang X., Chen H., Lei Y., Wang Q., Li G., Bai J. (2025). Association of Novel Mutations in the Vasoactive Intestinal Peptide Receptor-1 Gene with Egg Shell Thickness in Three Strains of Laying-Type Quail. Animals.

[10] Shaker A.S., Ameen Q.A., Beige M.M., Ortega Torres M., Alsalihi L.W. (2021). Using linear regression equation of egg dimensions in chicken to predict (Area, Volume, and Egg shape index). J. Kirkuk Univ. Agri. Sci..

[11] Xu H., Zeng H., Luo C., Zhang D., Wang Q., Sun L., Yang L., Zhou M., Nie Q., Zhang X. (2011). Genetic effects of polymorphisms in candidate genes and the QTL region on chicken age at first egg. BMC Genet..

[12] Promket D., Pengmeesri K., Kammongkun J., Somchan T. (2024). Identification of melatonin receptors type c (MTNR1C) and neuropeptide y (NPY) genes related to egg production in Thai indigenous chickens. Adv. Anim. Vet. Sci..

[13] Hosnedlova B., Vernerova K., Kizek R., Bozzi R., Kadlec J., Curn V., Kouba F., Fernandez C., Machander V., Horna H. (2020). Associations between IGF1, IGFBP2 and TGFß3 genes polymorphisms and growth performance of broiler chicken lines. Animals.

[14] Fu M., Wu Y., Shen J., Pan A., Zhang H., Sun J., Liang Z., Huang T., Du J., Pi J. (2023). Genome-wide association study of egg production traits in Shuanglian chickens using whole genome sequencing. Genes.

[15] Alig B.N., Malheiros R.D., Anderson K.E. (2023). Evaluation of physical egg quality parameters of commercial brown laying hens housed in five production systems. Animals.

[16] Darmawan A., Hermana W., Suci D.M., Mutia R., Jayanegara A., Ozturk E. (2022). Dietary phytogenic extracts favorably influence productivity, egg quality, blood constituents, antioxidant and immunological parameters of laying hens: A meta-analysis. Animals.

[17] Alfonso-Carrillo C., Benavides-Reyes C., de Los Mozos J., Dominguez-Gasca N., Sanchez-Rodríguez E., Garcia-Ruiz A.I., Rodriguez-Navarro A.B. (2021). Relationship between bone quality, egg production and eggshell quality in laying hens at the end of an extended production cycle (105 weeks). Animals.

[18] Li Z., Wu H., Fu J., Mushtaq M., Khan M., Liu Y., Azeem Z., Shi H., He Y., Zhang R. (2024). Eggshell Quality Traits and Transcriptome Gene Screening Between Yunnong and Jingfen Chicken Breeds. Biology.

[19] Saleh A.A., El-Awady A., Amber K., Eid Y.Z., Alzawqari M.H., Selim S., Soliman M.M., Shukry M. (2021). Effects of sunflower meal supplementation as a complementary protein source in the laying hen’s diet on productive performance, egg quality, and nutrient digestibility. Sustainability.

[20] Esenbuga N., Ekinci O. (2023). Dietary effects of some plant extracts on laying performance, egg quality, and some blood parameters in laying hens at different cage densities. Animals.

[21] Sun C., Qu L., Yi G., Yuan J., Duan Z., Shen M., Qu L., Xu G., Wang K., Yang N. (2015). Genome-wide association study revealed a promising region and candidate genes for eggshell quality in an F2 resource population. BMC Genom..

[22] Dunn I., Joseph N., Bain M., Edmond A., Wilson P., Milona P., Nys Y., Gautron J., Schmutz M., Preisinger R. (2009). Polymorphisms in eggshell organic matrix genes are associated with eggshell quality measurements in pedigree Rhode Island Red hens. Anim. Genet..

[23] Fleet J.C., Bruns M.E., Hock J.M., Wood R.J. (1994). Growth hormone and parathyroid hormone stimulate intestinal calcium absorption in aged female rats. Endocrinology.

[24] Alkan S., Karslı T., Durmuş İ., Karabağ K. (2016). Effects of egg shape index on egg quality in guinea fowl (Numida meleagris) [in Tukish]. Turk. J. Agric. -Food Sci. Technol..

[25] Alkan S., Türker İ. (2021). Effects of egg shape index on egg quality in partridges. Ordu Univ. J. Sci. Technol..

[26] Sekeroglu A., Kayaalp G., Sarıca M. (2000). The regression and correlation analysis an egg parameters in Denizli poultry. J. Agric. Fac. Cukurova Univ..

[27] Alasahan S., Copur A. (2016). Hatching characteristics and growth performance of eggs with different egg shapes. Rev. Bras. Ciência Avícola.

[28] Wu F., Gu L., Shang Y., Zhang X., Xu Z., Xu T. (2025). A novel 22-bp InDel in the intron 1 of the IGF1 gene is associated with slaughtering performance of Chinese Jiaji duck. Anim. Prod. Sci..

[29] Yang W., Yu S., Song D., Lin W., Xu H., Lang X., Zhang C., Guo L., Chen X. (2025). A genome-wide association study identified candidate genes associated with egg quality traits in Muscovy duck. BMC Genom..

[30] Dang D.X., Chung Y.H., Kim I.H. (2021). Effects of dietary supplementation of herbal active ingredients promoting insulin-like growth factor-1 secretion on production performance, egg quality, blood hematology, and excreta gas emission in laying hens. Anim. Biosci..

[31] Zhou M., Du Y., Nie Q., Liang Y., Luo C., Zeng H., Zhang X. (2010). Associations between polymorphisms in the chicken VIP gene, egg production and broody traits. Brit Poult. Sci..

[32] Amir M.J.A., Al-Anbari E.H., Razuki W.M. (2019). Polymorphism of VIP gene C+ 338T and its Association with the egg production of local Iraqi brown chicken. Biochem. Cell. Arch..

[33] Karslı T., Demir E., Fidan H.G., Karslı B.A., Aslan M., Aktan S., Kamanlı S., Karabağ K., Semerci E.Ş., Balcıoğlu M. (2020). Polymorphisms in candidate genes associated with egg yield and quality in brown layer pure lines. Mediterr. Agric. Sci..

[34] Nguyen T.T.B., Duc N.H., Quy V.C., Yen H.T., Loan T.T., Thuy D.T.N., Tien V.T., Thuy N.T.D. (2018). Effect of nucleotide polymorphism of candidate genes on egg production traits in native Lien Minh chicken. Livest. Res. Rural. Dev..

[35] Jun L., Yan L. (2013). Anti-bacterial activity of recombinant human β-defensin-3 secreted in the milk of transgenic goats produced by somatic cell nuclear transfer. PLoS ONE.

[36] Al-Zubaidi K., Al-Rekabi M., Allaw A. (2023). Effect of polymorphism of the Neuropeptide Y (NPY) gene on some productive traits of Iraqi local white chickens. IOP Conference Series: Earth and Environmental Science.

[37] Padwar P., Thakur M. (2021). Association of neuropeptide-Y gene polymorphic variants with quantitative traits in Jabalpur colour and Kadaknath chicken. Indian. J. Anim. Sci..

[38] Kammongkun J., Promket D. (2024). Growth performance and morphology traits associated with neuropeptide y (npy) genes expression in native chickens. Adv. Anim. Vet. Sci..

[39] Daş H., Tarim B., Demir S., Küçükkent N., Cengiz S., Tülek E., Aksakal V. (2017). Association of IGF and IGFBP2 gene polymorphisms with growth and egg traits in Atak-S laying hens. J. Hell. Vet. Med. Soc..

[40] Haugh R.R. (1937). The Haugh unit for measuring egg quality. US Egg Poult. Mag..

[41] Xiao X., Zhu Y., Deng B., Wang J., Shi S., Wang S., Han X., Zhao L., Song T. (2023). Effects of Dietary Phytosterol Supplementation on the Productive Performance, Egg Quality, Length of Small Intestine, and Tibia Quality in Aged Laying Hens. Animals.

[42] Jing X., Wang Y., Song F., Xu X., Liu M., Wei Y., Zhu H., Liu Y., Wei J., Xu X. (2022). A Comparison between Vitamin D3 and 25-Hydroxyvitamin D3 on Laying Performance, Eggshell Quality and Ultrastructure, and Plasma Calcium Levels in Late Period Laying Hens. Animals.

[43] Miller S.A., Dykes D.D., Polesky H. (1988). A simple salting out procedure for extracting DNA from human nucleated cells. Nucleic Acids Res..

[44] Thakur M., Parmar S., Chaudhari M., Bhardwaj J. (2009). Growth hormone gene polymorphism and its association with egg production in Kadaknath chicken. Livest. Res. Rural. Dev..

[45] Attarchi H., Tahmoorespur M., Ahani A.M., Sekhavati M.H., Mohajer M. (2017). Allelic polymorphism of GH, GHR and IGF-1 genes and their association with growth and carcass traits in Mazandaran native fowl. Poult. Sci. J..

[46] Wu P., Wang D., Jin C., Zhang X., Wu H., Zhang L., Ding F., Xie K., Zhang G. (2017). Polymorphisms of AluI and Hin1I loci of the IGF-1R gene and their genetic effects on growth traits in Bian chickens. Genet. Mol. Res..

